# An accelerated transgene-free genome editing system using microparticle bombardment of sorghum immature embryos

**DOI:** 10.1007/s42994-025-00204-9

**Published:** 2025-03-04

**Authors:** Yan Zhang, Ming Cheng, Karen Massel, Ian D. Godwin, Guoquan Liu

**Affiliations:** 1 School of Agriculture and Food Sustainability, The University of Queensland, St Lucia, 4072 Australia; 2 Plant Genetic Engineering Laboratory, School of Agriculture and Food Sustainability, The University of Queensland, St Lucia, 4072 Australia; 3 Centre for Crop Science, Queensland Alliance for Agriculture and Food Innovation, The University of Queensland, St Lucia, 4072 Australia

**Keywords:** *PDS* gene, Transgene-free, Genome editing, Sorghum transformation, CRISPR/Cas9, Transient gene expression

## Abstract

The key factors for genome-editing in plants using CRISPR/Cas9, such as the Cas9 nuclease and guide RNA (gRNA) are typically expressed from a construct that is integrated into the plant genome. However, the presence of foreign DNA in the host genome causes genetic and regulatory concerns, particularly for commercialization. To address this issue, we developed an accelerated pipeline for generating transgene-free genome-edited sorghum (*Sorghum bicolor*) in the T_0_ generation. For proof-of-concept, we selected the *Phytoene desaturase* (*PDS*) gene as the target due to its visible phenotype (albinism) upon mutation. Following microprojectile-mediated co-transformation with a maize (*Zea mays*)-optimized Cas9 vector and a guide RNA (gRNA) cassette with a geneticin (G418) resistance gene, we divided tissue derived from immature embryos into two groups (with and without antibiotic selection) and cultured them separately as parallel experiments. In regenerated plants cultured on medium containing MS basal nutrition (to allow albino plants to survive), we detected higher rates of albinism in the non-selection group, achieving editing rates of 11.1–14.3% compared with 4.2–8.3% in the antibiotic selection group. In the T_0_ generation, 22.2–38.1% of albino plants from the non-selection group were identified as transgene-free, whereas only 0–5.9% from the selection group were transgene-free. Therefore, our strategy efficiently produced transgene-free genome-edited plants without the need for self-crossing or outcrossing, demonstrating the feasibility of achieving transgene-free genome-edited sorghum plants within a single generation. These findings pave the way for commercializing transgene-free genome-edited lines, particularly for vegetatively propagated crops like pineapple, sugarcane, and banana.

**Supplementary Information:**

The online version contains supplementary material available at 10.1007/s42994-025-00204-9.

## Introduction

The clustered regularly interspaced short palindromic repeats (CRISPR)/CRISPR-associated nuclease 9 (Cas9) system has been widely used for plant genome editing (Ma et al. 2015; Kim and Kim 2019; Kumar et al. 2020) to investigate gene function, for plant molecular breeding, and for crop improvement (Zhang et al. 2021). The CRISPR/Cas9 system has tremendous advantages over traditional methods because it allows mutations to be easily generated in a precise and predictable manner (Kumar et al. 2020). The process of genome editing is typically based on stable genetic transformation, meaning that the two essential components of gene editing, Cas9 and the gRNA cassette, are expressed from a construct that is stably incorporated into the plant genome. However, apart from the selective marker that is also introduced, these foreign elements are no longer required once the target gene is completely edited by CRISPR/Cas9, which should occur soon upon transformation. Indeed, the continued presence of these components could cause off-target or continuous genome editing due to constitutive gene expression (Mao et al. 2019). Moreover, the presence of foreign DNA can cause regulatory complications that can hinder commercialization.

Global regulatory systems vary considerably but usually categorize plants containing foreign DNA as genetically modified organisms (GMOs) (Ahmad et al. 2021; Gupta et al. 2021). By contrast, gene-edited (GE) crops not containing foreign DNA are often subjected to less-stringent regulation. Genome-modified products can be classified into four groups according to the type of DNA double-strand break modification: the first group encompasses oligonucleotide-mediated (ODM) genome modifications, and the remaining three groups that plants are created by using site-directed nuclease (SDN) technologies including Zinc-finger nucleases, Transcription activator-like effector nucleases (TALENs), and CRISPR/Cas. Those three groups are detailed as SND1 (random mutation, short indels), SND2 (substitution or addition of a few nucleotides), and SND3 (the addition of large DNA fragments) (Zhang et al. 2021). In Australia, the Office of the Gene Technology Regulator classifies crops that were produced by SDN1 as non-GMOs and treats them the same as conventional crops, whereas crops generated using templates, which are classified as ODM, SDN2, or SDN3, are considered as GMOs (Ahmad et al. 2021). In Japan, SDN1 and SDN2 are treated as non-GMO crops. In the United States, ODM, SDN1, and SDN2 plants are deregulated (Wolt et al. 2016). However, plants in the SDN3 group are evaluated on a case-by-case basis for regulatory categorization (Ahmad et al. 2021). Transgene-free plants whose target genes were precisely manipulated by CRISPR/Cas9 genome editing would fall in the SDN1 classification and thus be treated as non-GMO crops by many countries. Therefore, the production of transgene-free genome-edited plants offers advantages over transgenic plants in streamlining the regulatory process for commercial production and alleviating public concerns.

Numerous strategies have been explored for generating transgene-free genome-edited plants, which can be categorized into three major approaches: (1) DNA-free gene editing, (2) transient expression of plasmid-based CRISPR-DNA, and (3) stable integration with self-crossing or subsequent backcrossing to eliminate the transgene. DNA-free gene editing techniques, including ribonucleoprotein (RNP) and virus-induced gene editing, have gained traction in genome editing for crops improvement (He et al. 2022; Li et al. 2024). Among these, preassembled CRISPR/Cas RNPs represent a direct, efficient approach for DNA-free genome editing. The application of preassembled CRISPR/Cas9 RNPs was pioneered in 2005, initially in Arabidopsis (*Arabidopsis thaliana*), tobacco (*Nicotiana tabacum*), lettuce (*Lactuca sativa*), and rice (*Oryza sativa*) protoplasts and subsequently in other plant species (Ahmad et al. 2021; Gupta et al. 2021).

Although the use of RNPs is prevalent, it presents challenges, particularly in economically vital crops with complex tissue culture systems. Among cereals, RNP has been successfully utilized to obtain transgene-free, homozygous mutant wheat (*Triticum aestivum*) in the T_0_ generation with frequencies of 0–6.8% (Zhang et al. 2016). In another report, the maize (*Zea mays*) mutation lines were not successfully generated in T_0_ generation; however, transgene-free mutant plants were obtained through RNP in the F_2_ progenies (Chen et al. 2018). Moreover, protoplast-based methods for genome editing, which are reported from many crops such as rice, tomato, pose challenges in recalcitrant crops such as sorghum (*Sorghum bicolor*) (Sairam et al. 1999). Similarly, efforts have been made to explore the use of alternative materials such as zygotes and immature embryos, although these alternatives remain limited to specific plant species (Wolt et al. 2016; He et al. 2022). Methods such as self-pollination and outcrossing have been used successfully to eliminate transgenes after gene editing, by identifying transgene-free lines through rigorous PCR screening and sequencing of T_1_ or T_2_ progenies (Chen et al. 2018; Li et al. 2020). However, these PCR-based methods are time-consuming and labour-intensive when identifying transgene-free plants from a pool of transgenic plants. To enhance efficiency, various markers have been investigated, such as fluorescent markers, pigments, chemicals, enzymatic reactions, and cell death genes (He et al. 2022).

Developing robust methodologies for eliminating or avoiding the integration of transgenes is crucial for facilitating the commercialization of genome-edited crops. Here, we report on a breakthrough in achieving transgene-free genome-edited sorghum plants, within a single generation, using particle bombardment. We used gene editing to target the phytoene desaturase (*PDS*) gene, encoding a critical enzyme in carotenoid biosynthesis to provide photo-protection for chlorophyll (Bartley and Scolnik 1995). Use of this gene allowed easy visualization of successful gene editing. This achievement is important for industrial applications in sorghum and holds promise for other monocot species.

## Results

### Design of a dual sgRNA system for efficient genome editing in sorghum

**Fig. 1:**
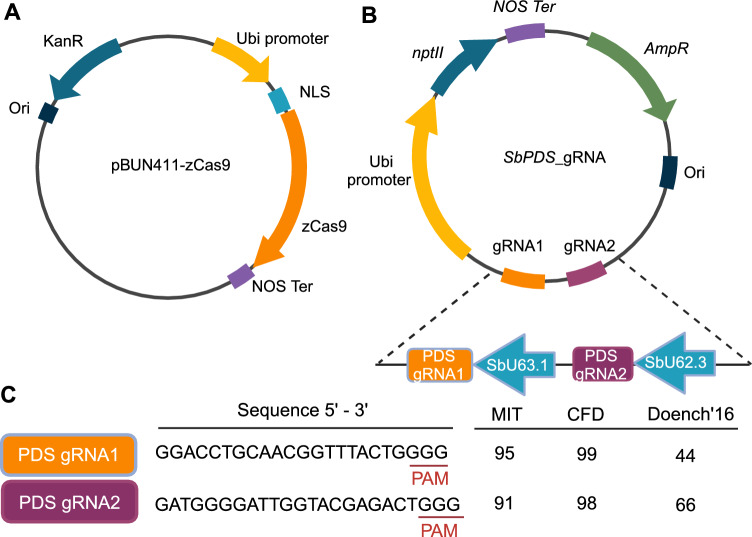
Schematic diagrams of the two plasmids used in this study. **A** zCas9 plasmid. **B** *SbPDS_*gRNA plasmid with two *PDS* gRNAs. **C** *PDS* gRNA sequences and the MIT, CFD, and Doench’16 scores returned by CRISPOR (Hsu et al. 2013; Doench et al. 2016; Concordet and Haeussler 2018)

To explore the possibility of generating transgene-free genome-edited sorghum plants via simple biolistic bombardment, we selected the *PDS* gene as our target for easy visualization of successful gene editing. We constructed two plasmids containing the critical elements for CRISPR/Cas9-mediated genome editing. The first plasmid, pBUN411-zCas9 (Fig. 1A), harbours the maize codon-bias optimized *Cas9* gene (*zCas9*) driven by the maize *ubiquitin* promoter (*Ubi*). The second plasmid, *SbPDS*_gRNA (Fig. 1B), includes two gRNAs targeting the sorghum *PDS* sequence (Fig. 1C) driven by two sorghum *U6* promoters: *SbU62.3* and *SbU63.1* (Fig. 1B) (Massel et al. 2022). gRNAs were designed using the web-based tool CRISPOR, with selection criteria including an on-target probability score and a GC content ranging from 40 to 70%. We chose gRNAs with relatively high MIT specificity scores (Hsu et al. 2013) and cutting frequency determination (CFD) specificity, as well as elevated Doench’16 efficacy scores (Doench et al. 2016); the latter was prioritized as the most critical parameter for selection. *SbPDS*_gRNA also contains the neomycin phosphotransferase (*NPTII*) gene, conferring resistance to a range of aminoglycoside antibiotics including geneticin (G418).

### Accelerated screening of putative genome-edited sorghum with targeted *PDS* genes

**Fig. 2:**
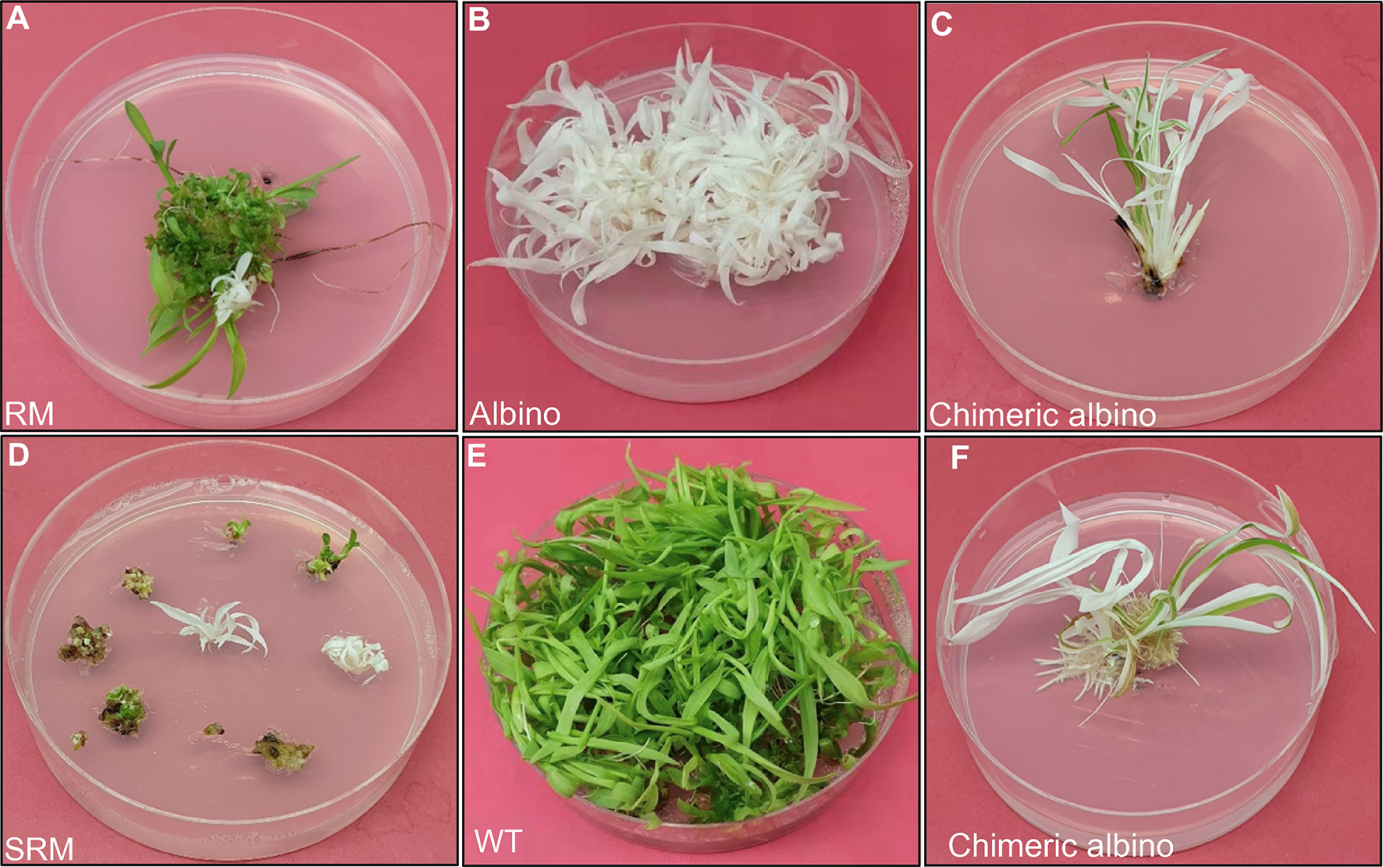
Phenotypes of wild-type and *PDS* mutant plantlets. **A** Regenerated plantlets on regeneration medium (RM) at 3 weeks post-bombardment. **B** Albino plants were isolated and cultured on RM for rapid growth; the photograph was taken at 8 weeks post-bombardment. **C** Chimeric albino plants on RM at 8 weeks post-bombardment. **D** Regenerated plantlets on selective regeneration medium (SRM) at 4 weeks post-bombardment. **E** Wild-type Tx430 grown on RM for 8 weeks. **F** Chimeric albino plantlets on rooting medium at 10 weeks post-bombardment

We co-transformed immature embryo (IE)-derived tissues of sorghum inbred line Tx430 with the two plasmids by particle bombardment. The plant materials were then divided into two groups for subculture on two types of media: 1) regeneration medium (RM) without antibiotic selection; and 2) selective regeneration medium (SRM) with 30 mg/L geneticin. Experiment 1 consisted of 72 plates harboring seven IE-derived tissues per plate. The materials on 18 of the 72 plates were subcultured on RM as the RM group, while the materials on the remaining 54 plates were subcultured on SRM as the SRM group. Each albino shoot (or shoots from same location) on RM or SRM (Fig. 2A, D) was considered as a single albino event and was transferred to a fresh RM plate without selection to ensure vigorous and healthy growth (Fig. 2B). The remaining tissues on SRM were maintained on SRM with continued subculture for up to 8 weeks.

Experiment 2 was conducted for experimental validation and optimization. Based on observations from Experiment 1, IE-derived tissues in the SRM group were placed on selection (SRM) for only 3 weeks to constrain non-transformed cell growth. In Experiment 2, 27 of 83 plates were subcultured on RM, accounting for 32.5% of the total plates bombarded (Table 1). The remaining material was subcultured on SRM for 3 weeks, followed by RM for the remaining subcultures. In both experiments, albino phenotypes were observed as early as 3 weeks post-bombardment in the SRM group with antibiotic selection (Fig. 2D). The emergence of albino phenotypes occurred approximately 4 weeks post-bombardment in the non-selection/RM group due to competition for nutrients among developing cells (Fig. 2A).

**Table 1:** Comparison of albino events between the two independent experiments

Batches	Total no. of plates	Group	No. of plates per group	Selection (weeks)	No. of IEs	Regenerated shoots	No. of albino events	Albino efficiency^a^ (%)	No. of transgene-free albinos
Exp1	72	RM	18	0	126	8366	18	14.3	4 (22.2%)
		SRM	54	3–8	378	146	16	4.2	0 (0%)
Exp2	83	RM	27	0	189	15,664	21	11.1	8 (38.1%)
		SRM	56	3	392	5983	34	8.7	2 (5.9%)

^a^Albino efficiency = No. of albino events regenerated/total number of IEs in each group × 100%

Upon their detection, albino individuals were separated and subcultured on a fresh RM plate (Fig. 2B, C). Wild-type sorghum line Tx430 was also subjected to regular tissue culture as a control (Fig. 2E). Although several chimeric albinos were observed (Fig. 2C, F), they were not included in calculations of editing or transformation efficiency due to the uncertain nature of chimeras.

In these experiments, we identified adequate levels of *PDS* gene editing leading to albino phenotypes. We obtained 18 albino plants (14.3%) from the RM group and 16 albino plants (4.2%) from the SRM group in Experiment 1. More IE-derived tissues were assigned to the RM and SRM groups in Experiment 2, leading to the production of 21 (11.1%) albinos from RM and 34 (8.7%) albinos from SRM (Table 1). Thus, the regeneration efficiency of albino sorghum plants (per IE) was 11.1–14.3% for the RM group but only 4.2–8.7% for the SRM group among all IEs bombarded.

The albino plants that were regenerated from both groups (with/without antibiotic selection) were large enough to be assessed and processed for genomic DNA extraction at approximately 10 weeks post-transformation. The entire process, from IE initiation to the assessment of albino plants, took only ~ 80 days (or 11.4 weeks). This is much shorter than conventional sorghum transformation systems, which take ~ 20 weeks; however, the use of morphogens can shorten the duration of conventional techniques to 10–12 weeks (Gurel et al. 2009). Therefore, our sorghum transformation scheme in which we targeted the *PDS* gene was comparable with conventional techniques for quickly generating genome-edited sorghum plants.

### Genotyping analysis of albinos via PCR screening

**Fig. 3:**
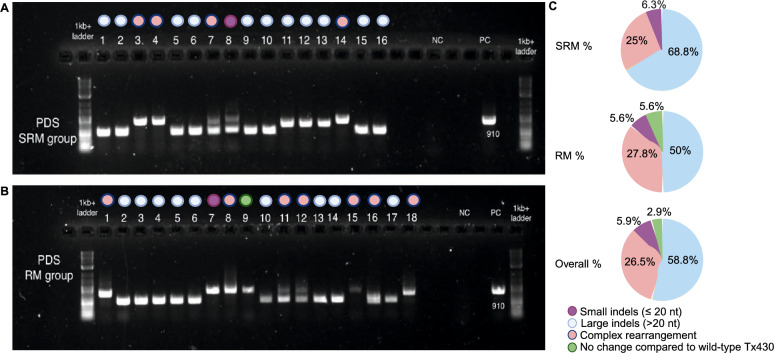
PCR analysis of the *PDS* gene in albino plants from groups SRM and RM in Experiment 1. **A** Results of PCR for the *PDS* gene in 16 albino plants from the SRM group. **B** Results of PCR for the *PDS* gene in 18 albino plants from the RM group. NC: No-template control. PC: plant control Tx430. The *PDS* gene fragment is 910 bp in PC. **C** Proportions of different editing events. The pie charts show the proportions of various editing events in the *PDS* gene in plants grown on SRM, RM, and the overall proportions of editing types

After we identified albino plants from the RM or SRM groups, we subcultured them individually on RM plates for rapid growth. Approximately 10 weeks after transformation, we genotyped the albino plants by PCR screening. When we amplified the *PDS* genes from each albino plant, we observed different sizes of PCR amplicons on agarose gels (Fig. 3A, B), indicating that different genome editing activities had occurred, such as large fragment deletions and potential biallelic edits. Figure 3C provides a comprehensive overview of the percentage distribution of different editing events. Based on subsequent analysis including Sanger sequencing, we categorized the editing events into four major groups: (1) small indels (no more than 20-nucleotide insertions or deletions), (2) large indels (> 20-nucleotide insertions or deletions), (3) complex rearrangements (mixture of deletions and insertions), and (4) no change (compared with wild-type Tx430, no editing was identified within the target PCR region). Among both groups, large fragment deletions emerged as the most frequent event due to the use of a dual sgRNA system, accounting for 58.8%, followed by complex rearrangements (26.5%) and small indels (5.9%) (Fig. 3C). Small indels (purple label in Fig. 3) with ≤ 20 nucleotide differences compared with wild-type Tx430 were relatively rare in both the SRM and RM groups. These indels accounted for 6.3% (1 out of 16 albino plants) in the SRM group and 5.6% (1 out of 18 albino plants) in the RM group. Only one albino plant from the RM group showed no difference in the target *PDS* region compared with wild-type Tx430; perhaps the mutation occurred outside the target region. We detected similar editing events in Experiment 2 (Online Resource Supplementary Table 1).

**Fig. 4:**
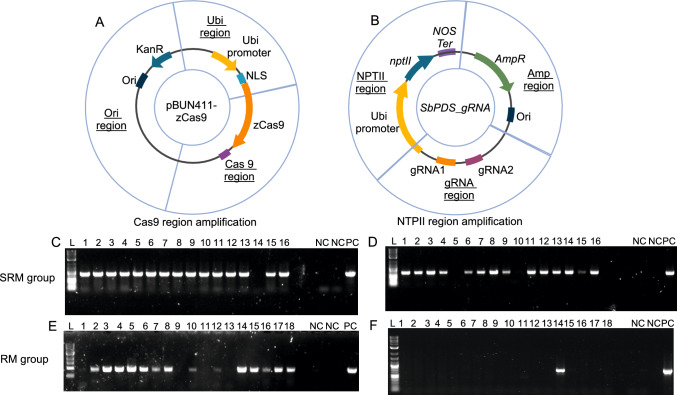
Regions subjected to PCR screening in Experiment 1. **A**, **B** The two plasmids were partitioned into distinct regions to examine the presence of transgene elements. In the zCas9 plasmid, we focused on the *Ubi*, *Cas9*, and *Ori* regions, whereas in the *SbPDS*_gRNA plasmid, we focused on the *NPTII*, *Amp*, and gRNA scaffold regions. **C–F** PCR results showing the amplification of *Cas9* and *NPTII* regions from 16 albino plants in the SRM group and 18 albino plants in the RM group in Experiment 1. L: DNA ladder, NC: No-template control, PC: Plasmid control

To confirm the transgenic status of albino plants, we performed PCR using six primer pairs targeting distinct regions of the zCas9 and *SbPDS*_gRNA plasmids to account for fragmented integration (Fig. 4A, B); the results for both experiments are summarized in Online Resource Supplementary Table 2. Albino plants lacking any of the six PCR regions were identified as transgene-free genome-edited plants. Most albino plants displayed a PCR-positive band of *Cas9*, indicating the presence of a *Cas9* region in their genomes regardless of the selection strategy (Fig. 4C, E). By contrast, the presence of the selective marker (*NPTII*) was different for plants in the SRM group (Fig. 4D) vs. the RM group (Fig. 4D, F). Notably, most albino samples in the SRM group contained the *NPTII* gene due to consistent selection pressure. Albino SPDSM-5 and SPDSM-10 (the first S stands for selection from the SRM groups and M stands for mutant) did not show the *NPTII* PCR band, likely due to early release from selection. When albino plantlets appeared, we transferred the corresponding shoots to RM without selection to promote shoot growth. This shortened selection process led to some escapes not containing the *NPTII* selective marker. The genome editing of these albino plants was attributed to transient expression of *Cas9* and *NPTII*. SPDSM-14 contained *NPTII* but lacked *Cas9* integrated into its genome, which was also likely due to transient expression of *Cas9* and *NPTII*.

In the RM group, we did not detect the *NPTII* PCR band in most albino plantlets; this is understandable, as no selection was applied to this group, and therefore there was no selection pressure for *NPTII* integration. However, PDSM-14 exhibited the *NPTII* band, suggesting that without selection pressure, there is still a small chance for stable integration of exogenous genes into the plant genome (Fig. 4). Many of the samples still showed evidence for the integration of *Cas9* into the genome. Among the albino samples PDSM-1, PDSM-9, PDSM-11, and PDSM-13, neither *Cas9* nor *NPTII* was integrated into the genome, indicating that transient gene expression is sufficient for genome editing. The PCR results for the remaining plasmid regions are shown in Online Resource Supplementary Table 2. The four samples from the RM group in Experiment 1 were identified as transgene-free genome-edited plantlets. Similarly, eight other albino plantlets from the RM group in Experiment 2 were identified as transgene-free genome-edited plantlets (Online Resource Supplementary Table 2).

Overall, there was a higher prevalence of *NPTII* bands in the SRM vs. RM groups. This observation underscores the potential of the selection-free system for facilitating the generation of non-GM editing events. Sequencing confirmed that the targeted *PDS* gene was edited by CRISPR/Cas9 in albino plantlets in both the RM and SRM groups, as described below.

### Validation of CRISPR/Cas9-mediated editing events through Sanger sequencing

**Fig. 5:**
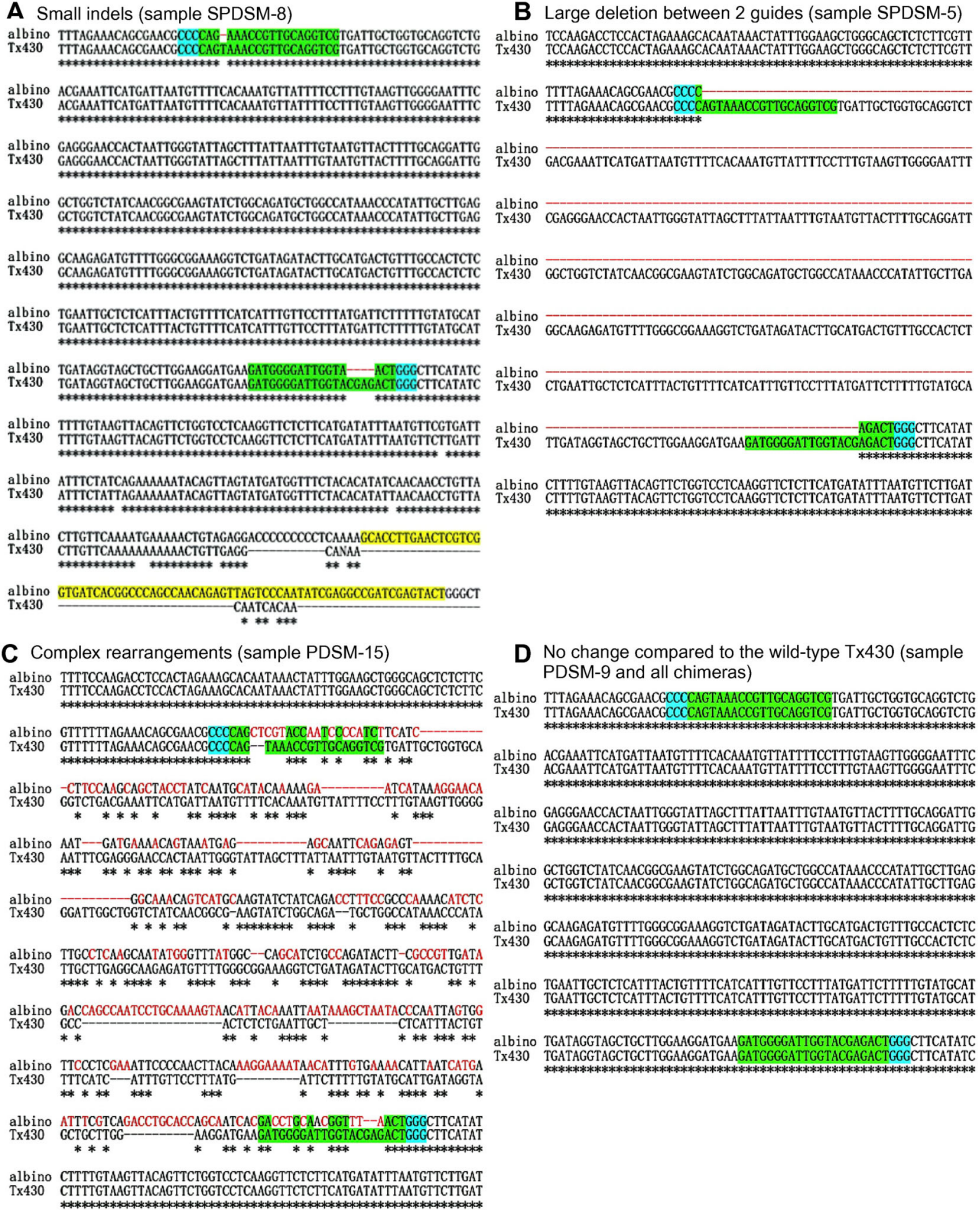
Examples of editing types in albino plants. **A** Small indels (sample SPDSM-8): one nucleotide lost in the first gRNA target displayed biallelic editing, which matches the PCR results showing double bands. Part of the *Cas9* plasmid (highlighted in yellow) was inserted after the second gRNA target. **B** Large deletion between two gRNA targets (SPDSM-8). **C** Complex rearrangements included multiple indels, and substitutions between the two gRNA targets (PDSM-15). **D** No change compared with wild-type Tx430. Green: binding sites of the gRNAs; Blue: PAM sequence; Red: positions of single nucleotide polymorphisms (SNPs) or CRISPR/Cas9-associated mutations occurring between the gRNA targets; Yellow: sequence matched up with the *cas9* plasmid

Across albino plantlets, Sanger sequencing revealed four distinct editing outcomes, as described above. A comprehensive summary detailing the frequency of each of these editing events is provided in Fig. 3C and Online Resource Supplementary Table 1. Figure 5 shows the sequences of selected examples representing the four different types of editing events compared with wild-type Tx430.

Most of the editing events were characterized by large fragment deletions and complex rearrangements, both orchestrated by dual gRNAs (Fig. 5B, C). Figure 5A shows an example of small indels with ≤ 20 base pair differences from wild-type Tx430. This study highlights the notion that the most frequent editing events typically involve large indels of > 20 base pairs (Online Resource Supplementary Table 1).

### RT-PCR of *Cas9* gene expression in albino plantlets

**Fig. 6:**
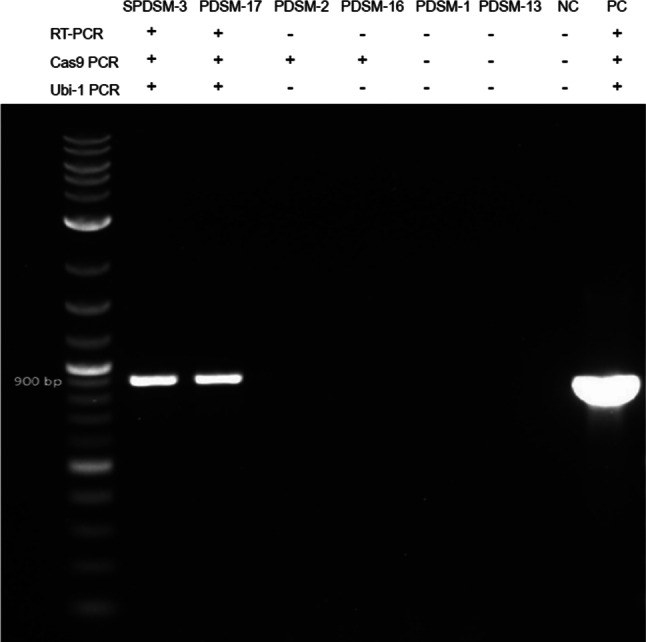
Expression analysis of *Cas9* by RT-PCR. From left to right: DNA marker: 1 kb + ladder; samples SPDSM-3 and PDSM-17 (with both *Cas9* and *Ubi* in their genomes) exhibited the *Cas9* gene expression after reverse transcription; samples PDSM-2 and PDSM-16 (with *Cas9* but without the *Ubi* promoter in their genomes) was not detected the C*as9* transcription by RT-PCR; samples PDSM-1 and PDSM-13 (without both C*as9* and *Ubi* in their genomes) was not detected the C*as9* transcription by RT-PCR; NC: no-template control; PC: zCas9 plasmid control

We employed *Ubi* primers designed against the *Cas9* plasmid to gauge the effectiveness of the *Ubi* promoter in driving *Cas9* expression. Based on the *Cas9* amplification results shown in Fig. 4 and Online Resource Supplementary Table 2, we examined *Cas9* expression in detail. We selected six albino plants for reverse transcription PCR (RT-PCR) analysis, each with distinct DNA integration patterns: SPDSM-3 and PDSM-17 exhibited both *Cas9* and *Ubi* fragments, PDSM-2 and PDSM-16 contained *Cas9* but lacked the *Ubi* fragment, and PDSM-1 and PDSM-13 lacked both *Cas9* and *Ubi*. As shown in Fig. 6, we detected successful *Cas9* expression only in SPDSM-3 and PDSM-17. These RT-PCR results aligned with the PCR results described above, reinforcing the efficiency of the *Ubi* promoter in driving *Cas9* transcription. These results demonstrate that the *Cas9* gene and its promoter (*Ubi*) are indispensable for *Cas9* expression in cells (Fig. 6).

### Comparison of the results of replicate experiments

Based on the results of Experiment 1, we assigned a greater proportion of bombarded plates to the RM group in Experiment 2 and maintained selection in the SRM group for only 3 weeks in an effort to obtain more transgene-free genome-edited albino sorghum plants. This adjustment allowed for a more robust comparison of the production of albino plants between two groups with or without selection pressure.

**Fig. 7:**
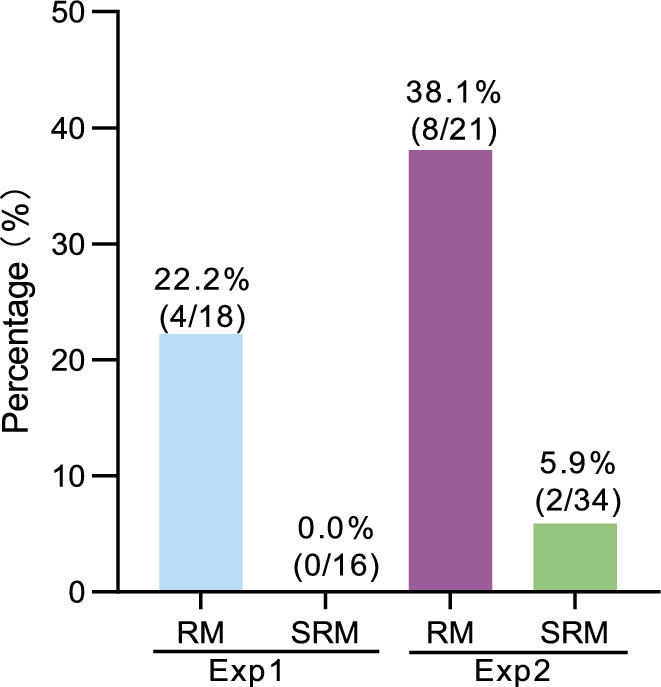
Frequencies of transgene-free genome editing in two experiments. The frequency is shown as the number of transgene-free genome-edited albino plants divided by the total number of albino plants obtained from each group in each experiment. The number of transgene-free genome-edited plants and the number of albino plants are shown in parentheses. For example, for the RM group in Experiment 1, four transgene-free genome-edited plants were identified among 18 albino plants

The results from Experiment 2 were consistent with those from Experiment 1. In the RM group, the number of regenerated plants in Experiment 2 (15,664) was nearly double that in Experiment 1 (8366). The number of regenerated non-transgenic albino events was also double, with eight transgene-free genome-edited albino plants in Experiment 2 compared with four in Experiment 1. We produced transgene-free genome-edited plants at a frequency of 22.2% in Experiment 1 and 38.1% in Experiment 2 (Fig. 7).

In the SRM groups, a similar number of IEs and plates were assigned to both experiments. We optimized the selection procedure by transferring albino calli to regeneration medium as soon as they were detected on selection plates. This led to a higher frequency of albino plant production, reaching 8.7% in Experiment 2 compared with 4.2% in Experiment 1 (Fig. 7). Ultimately, two transgene-free genome-edited albino plants (2 out of 34) were obtained from the SRM group in Experiment 2 since these materials were subjected to selection for only 3 weeks, resulting in a rate of 5.9%. Overall, the results from Experiment 2 suggest that using a greater number of plates in the RM group could lead to the production of more non-transgenic plants. Further optimization of the selection method might also facilitate the regeneration of non-transgenic plants, which could be modified to increase the number of high-quality events for transgene-free gene editing.

## Discussion

In this study, we employed particle bombardment for gene editing in sorghum plants with the aim of establishing a method for transgene-free genome editing. Due to the phenotypic change (albinism) caused by homozygous/biallelic *PDS* mutations, putative genome-edited plants were easy to detect for subsequent downstream analysis. Remarkably, 22.2% and 38.1% of homozygous/biallelic edited lines in the non-selection (RM) group in two individual experiments were identified as transgene-free lines in the T_0_ generation, with no traceable transgenes via PCR testing. This strategy could speed up the process of generating transgene-free edited plantlets, saving valuable time often used to eliminate transgene elements by self-crossing or outcrossing. Furthermore, our method provides an avenue for achieving transgene-free genome editing of vegetatively propagated crops.

The sorghum inbred line Tx430 is amenable to tissue culture, with an extremely low frequency of regenerating somaclonal albino plantlets throughout transformation and regeneration. In this study, only homozygous or biallelic edited albino plantlets were considered as successfully gene edited events, as we focused on saving time, effort, and costs by targeting the *PDS* gene, followed by visual assessment. These edits were verified through PCR and Sanger sequencing to assess the efficiency and types of editing outcomes in sorghum. In total, we obtained 89 albino plants, 83 of which were confirmed to be edited at the targeted *PDS* gene by CRISPR/Cas9-mediated genome editing. To identify transgene integration, we examined six regions by PCR, including *Ubi*, *Cas9*, *NPTII*, *Amp*, and *gRNA* (Fig. 4); plants in which we could not detect these plasmid fragments were regarded as non-transgenic events. Of the albino plants from the group without selection, ~ 30.8% (12 out of 39 albino plants) were determined to be transgene-free, whereas ~ 4% (2 out of 50 albino plants) were transgene-free following culture on SRM for at least 3 weeks post-bombardment.

As shown in Table 1, more *Cas9* fragments than *NPTII* fragments were integrated into the plant genome, suggesting that CRISPR/Cas9 editing generally requires the consistent presence of Cas9 protein from stable *Cas9* gene expression. Since we employed two sgRNAs targeting the *PDS* gene in sorghum, a relatively high level of DNA-sequence drop-out was expected between the two cut sites. Overall, small indels accounted for 5.9% mutation while large indels accounted for 58.8% mutation. In the current study, PCR and sequencing were efficient for identifying and characterizing genome editing events with known homozygous/biallelic outcomes.

Surprisingly, two albino plants (SPDSM-5 and SPDSM-10) lacked the *NPTII* gene from the SRM groups. This phenomenon, which is similar to transgene-free editing without antibiotic selection, could be attributed to the efficient transient expression of the selective marker gene in the cells of these plants, allowing them to grow under antibiotic selection for some time. In previous study, transient gene expression can last more than 10 days post-bombardment (Liu and Godwin 2012). As described in the Materials and Methods, during the subculture process, any albino plantlets identified on SRM or RM plates were transferred onto non-selection RM to accelerate their growth by reducing nutritional competition from the culture medium.

In future experiments where target genes lead to less obvious phenotypes, the identification of homozygous/biallelic plants will increase the time needed for screening. However, this could be reduced by pooling samples, followed by next-generation sequencing to identify any potential editing in the population. The rates of heterozygous editing in sorghum range from 0 to 40% (Massel et al. 2022). In experiments where it is essential to monitor heterozygous editing, such events could be distinguished based on sequencing results, where the peaks near the PAM (Protospacer Adjacent Motif) sequence can be different from those of wild-type plants (Liu et al. 2019). In the current study, more albino plants were generated under non-selection than under antibiotic selection pressure. In the absence of selection, transient gene expression has the chance to occur, sometimes leading to genome editing. Similar events were previously observed in wheat, where a slightly higher percentage of mutants was obtained in the non-selection groups when the *TaGW2* gene was targeted (Zhang et al. 2016).

The efficacy of genome editing is strongly affected by the design of the CRISPR/Ca9 genome editing system, and there is always room for improvement. Parameters to explore include the design of the plasmid DNA, the choice of an endogenous promoter to express gRNA, and the design of the gRNA. Our experiments demonstrate the feasibility of producing transgene-free genome-edited sorghum in one generation, but future experiments should explore the use of one plasmid containing all essential elements for gene editing. For gRNA design, we employed CRISPOR, where higher specificity scores generally correlate with fewer off-target sites and lower frequencies of off-target modifications, although new tools are always being developed and tested for their in vivo accuracy. In the future, potential off-target sites for specific gRNAs could be examined, if necessary. Moreover, a cytosine immediately following the PAM sequence is detrimental to genome editing (Bruegmann and Deecke 2019). Rather than using the *PDS* gene, other methods could aid in selecting transgene-free genome-edited sorghum plants, such as *RUBY*, a reporter that converts tyrosine into bright red betalain, which is visible to the naked eye without the need for special tools or chemical treatments (He et al. 2020; Yu et al. 2023; Chen et al. 2024). Such markers could be used to help selectively outcross transgene-positive plants with relative ease while maintaining gene-edited products. Furthermore, perhaps transformation efficiencies could be improved using morphogenic regulators (Che et al. 2022).

Overall, our study represents a significant advance in generating transgene-free genome-edited sorghum plants in a single generation via particle bombardment. Subsequent analysis and trials will undoubtedly require the detection of entire plasmids in plants. Whole-genome sequencing, which has become more affordable and streamlined, could be used to verify candidate transgene-free genome-edited events for commercialization in the future. The elimination of transgenes is pivotal for advancing the commercialization of genome-edited sorghum lines and has broader applications for other monocot and vegetatively propagated crops.

## Materials and methods

### Plant materials

The wild-type *Sorghum bicolor* Tx430 line was cultivated in the greenhouse at the University of Queensland using potting mix which consists of two major components (70% composted pine bark 0–5 mm and 30% coco peat), with a controlled temperature maintained at approximately 27–28 °C during the day and 20–22 °C at night. Although the greenhouses were temperature-controlled, the plants exhibited optimal health during the summer months, yielding the most suitable explants for tissue culture due to the elevated levels of sunlight. Regular applications of fertilizers were administered to the soil, and pest and disease surveillance was conducted weekly.

### Plasmids for genome editing

Two plasmids were used for sorghum transformation. pBUN411-zCas9 (Fig. 1A) harbours the maize code-optimized *Cas9* gene (*zCas9*) and was designed for effective use in *Agrobacterium*-mediated maize transformation (Xing et al. 2014). The *SbPDS*_gRNA vector (Fig. 1B), synthesized by Gene Universal (USA), includes two gRNAs designed to target the sorghum *PDS* sequence. Additionally, the vector contains the neomycin phosphotransferase gene (*NPTII*), providing resistance to a range of aminoglycoside antibiotics, including kanamycin and G418. The maize *ubiquitin* promoter was used to drive both *zCas9* and *NPTII* expression*.*

### Design of CRISPR gRNAs for the sorghum *PDS* gene

The gRNAs for the sorghum *PDS* gene (Sobic.006G232600, Alias: Sb06g030030) were designed using the online tools CRISPOR (crispor.tefor.net) and CRISPR-P 2.0 (Haeussler et al. 2016; Liu et al. 2017), which employ various grading systems represented as scores. These include the Fusi/Doench’16 score (recommended for the *U6* promoter), the MIT score (for guide specificity), and the CFD specificity score, which enhances accuracy in predicting off-target effects. Two gRNAs with high MIT and CFD scores (over 90) and relatively high Fusi/Doench scores were selected (listed in Fig. 1C). The optimal gRNAs for *PDS* were selected according to the guidelines of the online tools. G was added to the 5′ end of the gRNA to improve U6-mediated transcription (Lone et al. 2018).

### Sorghum transformation via biolistic bombardment and regeneration process

Sorghum tissue culture and transformation were performed as described by Liu et al. (2014). Briefly, immature embryos (IEs) were induced from disinfected immature seeds of sorghum inbred line Tx430. The two plasmids, zCas9 and *SbPDS*_gRNA, were coated onto 0.6-µm gold particles and delivered into 6–8 IE-derived tissues via particle bombardment. The transformed IE-derived calli were incubated on RM (MS medium supplemented with 1 mg/L BAP, 1 mg/L IAA, and 1 μM CuSO_4_) for 3 days after bombardment and divided into two groups: the SRM group was placed on RM containing 30 mg/L geneticin (G418), and the RM group was placed on RM with no selection. Albino phenotypes were observed as early as 3 weeks post-bombardment in the SRM group and at approximately 4 weeks post-bombardment in the RM group. Albino individuals were separated upon detection and subcultured on a fresh regeneration plate without selection. Once the albino plants were 3–5 cm tall, they were transferred to rooting medium (MS medium with 1 mg/L NAA, 1 mg/L IAA, 1 mg/L IBA, and 1 μM CuSO_4_). In general, albino plants regenerated from both groups (with/without antibiotic selection) were large enough to be assessed and processed for genomic DNA extraction at 10 weeks post-transformation. The entire process, from IE initiation to the assessment of albino plants, took ~ 80 days (or ~ 11.4 weeks).

### Design of PCR primers

Multiple regions of each plasmid, including the primary genes *zCas9* and *NPTII*, promoter (maize *ubiquitin* primer), and vector backbone (ampicillin-resistance gene and the Ori region), were targeted for PCR screening. All primers were designed using the online tool Primer3 (Untergasser et al. 2012) (Table 2).

**Table 2:** Primers targeting different regions in the two plasmids

Name	Sequence	Size (bp)	Target plasmid/gene	Annealing temperature (°C)
AmpF	CTGGCGTAATAGCGAAGAGG	746	*SbPDS_gRNA*	65.5
AmpR	ATAATACCGCGCCACATAGC			
KanF	AGACAATCGGCTGCTCTGAT	740	*SbPDS_gRNA*	65.5
KanR	TCATTTCGAACCCCAGAGTC			
gRNAF	GGGCGACGTTGTTTAGTACC	879	*SbPDS_gRNA*	64
gRNAR	GCACTTCAAACAAGTGTGACAA			
cPDSF	GGGCAGCTCTCTTCGTTTTT	477	*PDS*	60
cPDSR	TGAAGAGAACCTTGAGGACCA			
cPDSF2	ACACTGGCTGCCTCTCATCT	910	*PDS*	60
cPDSR2	GTGTGATGGTGTGCCTCAAC			
UbiF	GGGCCCGGTAGTTCTACTTC	875	*zCas9*	63.5
UbiR	CGTATGAAGGCAGGGCTAAA			
zCas9F	ACGGTTAAGCAGCTCAAGGA	900	*zCas9*	60
zCas9R	CCTGGTGAGGACCTTGTTGT			
OriF	CTGGCAGTTCCCTACTCTCG	1105	*zCas9*	67
OriR	GCCTACATACCTCGCTCTGC			

### DNA extraction from primary albino plantlets

Once the albino materials grew multiple shoots (5 cm long) on regeneration or rooting medium, leaf samples were collected from the albino shoots. Genomic DNA was extracted from young leaves using an ISOLATE II Plant DNA kit (Bioline, UK). The DNA concentration and quality were verified using a Thermo Scientific™ NanoDrop™ One (Thermo Fisher Scientific Inc., USA), as indicated by an A_260_/A_280_ absorbance ratio > 1.80. To check genomic DNA quality, 5 µL DNA was loaded onto a 1% (w/v) agarose-TAE gel with SYBR Safe (Invitrogen, USA) and subjected to gel electrophoresis at 90 V for 50 min; a 1 kb + DNA ladder (New England Biolabs, UK) was used as a DNA size indicator. A Gel Doc + Gel Documentation System (BIO-RAD, USA) was used to visualize the gel and document an image for observing the existence and size of the target products. The *PDS* PCR products were cleaned using ExoI and rSAP PCR Product Clean-up Reagent (New England Biolabs, UK) following the manufacturer’s protocol and sent to the Australian Genome Research Facility for Sanger sequencing using either the forward or reverse primer of the *PDS* gene.

### RNA extraction and reverse transcription PCR (RT-PCR)

To confirm *Cas9* gene expression in selected albino lines, total RNA was extracted from leaves using an ISOLATE II RNA Plant kit (Bioline, UK). When plants in rooting medium were ~ 5 cm tall, with multiple leaves, ~ 100 mg of fresh leaf tissue from each plant was ground in liquid nitrogen using a mortar and pestle, transferred into a new 1.5-mL tube, immediately mixed with lysis buffer (RLY), and subjected to RNA extraction following the manufacturer’s protocol. The concentration and quality of the extracted RNA were evaluated using a Thermo Scientific™ NanoDrop™ One (Thermo Fisher Scientific Inc., USA), as indicated by an A_260_/A_280_ absorbance ratio of ~ 2.1, followed by gel electrophoresis to detect clear 18S and 28S bands.

After verifying RNA quality, a GoScript™ Reverse Transcription System kit (Promega, USA) was used to synthesize first-strand cDNA according to the manufacturer’s instructions. Total RNA (1 µg) was incubated with 0.5 µg Oligo (dT)15 primer, and 3.75 mM MgCl_2_ was used to prepare the RT reaction mix. The first-strand cDNA was subjected to PCR with *zCas9* primers, and 2 µL cDNA was used in each reaction. The PCR products were separated by electrophoresis in 1% (w/v) agarose-TAE gels with SYBR Safe (Invitrogen, USA) at 90 V for 50 min.

## Supplementary Information

Below is the link to the electronic supplementary material.

Supplementary file1 (DOCX 39 KB)

## Data Availability

Data supporting this study are located in the Online Resource Supplementary Materials.

## References

[CR1] Ahmad AMZ, Ghouri NM et al (2021) Regulatory, ethical, and social aspects of CRISPR crops. In: CRISPR crops, pp 261–287. 10.1007/978-981-15-7142-8_9

[CR2] Bartley GE, Scolnik PA (1995) Plant carotenoids—pigments for photoprotection, visual attraction, and human health. Plant Cell 7(7):1027–1038. 10.1105/tpc.7.7.1027 7640523 10.1105/tpc.7.7.1027 PMC160905

[CR3] Bruegmann TK, Deecke MF (2019) Evaluating the efficiency of gRNAs in CRISPR/Cas9 mediated genome editing in Poplars. Int J Mol Sci 20(15):3623. 10.3390/ijms20153623 31344908 10.3390/ijms20153623 PMC6696231

[CR4] Che P, Wu E, Simon MK et al (2022) Wuschel2 enables highly efficient CRISPR/Cas-targeted genome editing during rapid de novo shoot regeneration in sorghum. Commun Biol 5(1):344. 10.1038/s42003-022-03308-w 35410430 10.1038/s42003-022-03308-w PMC9001672

[CR5] Chen RR, Xu QL, Liu Y et al (2018) Generation of transgene-free maize male sterile lines using the CRISPR/Cas9 system. Front Plant Sci 9:1180. 10.3389/fpls.2018.01180 30245698 10.3389/fpls.2018.01180 PMC6137208

[CR6] Chen L, Cai Y, Liu X et al (2024) The RUBY reporter for visual selection in soybean genome editing. aBIOTECH 5(2):209–213. 10.1007/s42994-024-00148-6 38974868 10.1007/s42994-024-00148-6 PMC11224211

[CR7] Concordet JP, Haeussler M (2018) CRISPOR: intuitive guide selection for CRISPR/Cas9 genome editing experiments and screens. Nucleic Acids Res 46(W1):W242–W245. 10.1093/nar/gky354 29762716 10.1093/nar/gky354 PMC6030908

[CR8] Doench JG, Fusi N, Sullender M et al (2016) Optimized sgRNA design to maximize activity and minimize off-target effects of CRISPR-Cas9. Nat Biotechnol 34(2):184–191. 10.1038/nbt.3437 26780180 10.1038/nbt.3437 PMC4744125

[CR9] Gupta S, Kumar A, Patel R et al (2021) Genetically modified crop regulations: scope and opportunity using the CRISPR-Cas9 genome editing approach. Mol Biol Rep 48(5):4851–4863. 10.1007/s11033-021-06477-9 34114124 10.1007/s11033-021-06477-9

[CR10] Gurel S, Gurel E, Kaur R et al (2009) Efficient, reproducible *Agrobacterium*-mediated transformation of sorghum using heat treatment of immature embryos. Plant Cell Rep 28(3):429–444. 10.1007/s00299-008-0655-1 19115059 10.1007/s00299-008-0655-1

[CR11] Haeussler M, Schonig K, Eckert H et al (2016) Evaluation of off-target and on-target scoring algorithms and integration into the guide RNA selection tool CRISPOR. Genome Biol 17:1–12. 10.1186/s13059-016-1012-2 27380939 10.1186/s13059-016-1012-2 PMC4934014

[CR12] He Y, Zhang T, Sun H et al (2020) A reporter for noninvasively monitoring gene expression and plant transformation. Hortic Res 7(1):152. 10.1038/s41438-020-00390-1 33024566 10.1038/s41438-020-00390-1 PMC7502077

[CR13] He YB, Mudgett M, Zhao YD (2022) Advances in gene editing without residual transgenes in plants. Plant Physiol 188(4):1757–1768. 10.1093/plphys/kiab574 34893903 10.1093/plphys/kiab574 PMC8968301

[CR14] Hsu PD, Scott DA, Weinstein JA et al (2013) DNA targeting specificity of RNA-guided Cas9 nucleases. Nat Biotechnol 31(9):827–832. 10.1038/nbt.2647 23873081 10.1038/nbt.2647 PMC3969858

[CR15] Kim JI, Kim JY (2019) New era of precision plant breeding using genome editing. Plant Biotechnol Rep 13(5):419–421. 10.1007/s11816-019-00581-w

[CR16] Kumar K, Gambhir G, Dass A et al (2020) Genetically modified crops: current status and future prospects. Planta 251(4):27. 10.1007/s00425-020-03372-8 10.1007/s00425-020-03372-8 32236850

[CR17] Legislation FR (2016) Gene technology regulations 2001. A. Government. https://www.legislation.gov.au/Details/F2016C00615

[CR18] Li CY, Li W, Zhou ZH et al (2020) A new rice breeding method: CRISPR/Cas9 system editing of the promoter to cultivate transgene-free bacterial blight-resistant rice. Plant Biotechnol J 18(2):313–315. 10.1111/pbi.13217 31344313 10.1111/pbi.13217 PMC6953186

[CR19] Li B, Sun C, Li J et al (2024) Targeted genome-modification tools and their advanced applications in crop breeding. Nat Rev Genet. 10.1038/s41576-024-00720-2 38658741 10.1038/s41576-024-00720-2

[CR20] Liu GQ, Godwin ID (2012) Highly efficient sorghum transformation. Plant Cell Rep 31(6):999–1007. 10.1007/s00299-011-1218-4 22234443 10.1007/s00299-011-1218-4 PMC3351618

[CR21] Liu GQ, Campbell BC, Godwin ID (2014) Sorghum genetic transformation by particle bombardment. Cereal Genomics Methods Protoc 1099:219–234. 10.1007/978-1-62703-715-0_18 10.1007/978-1-62703-715-0_18 24243207

[CR22] Liu H, Ding Y, Zhou Y et al (2017) CRISPR-P 2.0: an improved CRISPR-Cas9 tool for genome editing in plants. Mol Plant 10(3):530–532. 10.1016/j.molp.2017.01.003 28089950 10.1016/j.molp.2017.01.003

[CR23] Liu G, Li J, Godwin ID (2019) Genome editing by CRISPR/Cas9 in sorghum through biolistic bombardment. Methods Mol Biol 1931:169–183. 10.1007/978-1-4939-9039-9_12 30652290 10.1007/978-1-4939-9039-9_12

[CR24] Lone BA, Karna SKL, Ahmad F et al (2018) CRISPR/Cas9 system: a bacterial tailor for genomic engineering. Genet Res Int 2018:3797214. 10.1155/2018/3797214 30319822 10.1155/2018/3797214 PMC6167567

[CR25] Ma XL, Zhang QY, Zhu QL et al (2015) A robust CRISPR/Cas9 system for convenient, high-efficiency multiplex genome editing in monocot and dicot Plants. Mol Plant 8(8):1274–1284. 10.1016/j.molp.2015.04.007 25917172 10.1016/j.molp.2015.04.007

[CR26] Mao YF, Botella JR, Liu YG et al (2019) Gene editing in plants: progress and challenges. Natl Sci Rev 6(3):421–437. 10.1093/nsr/nwz005 34691892 10.1093/nsr/nwz005 PMC8291443

[CR27] Massel K, Lam Y, Hintzsche J et al (2022) Endogenous U6 promoters improve CRISPR/Cas9 editing efficiencies in *Sorghum bicolor* and show potential for applications in other cereals. Plant Cell Rep 41(2):489–492. 10.1007/s00299-021-02816-z 34854968 10.1007/s00299-021-02816-z

[CR29] Sairam RV, Seetharama N, Devi PS et al (1999) Culture and regeneration of mesophyll-derived protoplasts of sorghum [*Sorghum bicolor* (L.) *Moench*]. Plant Cell Rep 18(12):972–977. 10.1007/s002990050693

[CR30] Untergasser A, Cutcutache I, Koressaar T et al (2012) Primer3-new capabilities and interfaces. Nucleic Acids Res 40(15):12. 10.1093/nar/gks596 10.1093/nar/gks596 PMC3424584 22730293

[CR31] Wolt JD, Wang K, Yang B (2016) The regulatory status of genome-edited crops. Plant Biotechnol J 14(2):510–518. 10.1111/pbi.12444 26251102 10.1111/pbi.12444 PMC5042095

[CR32] Xing HL, Dong L, Wang ZP et al (2014) A CRISPR/Cas9 toolkit for multiplex genome editing in plants. BMC Plant Biol 14:12617. 10.1038/ncomms12617 10.1186/s12870-014-0327-y PMC4262988 25432517

[CR33] Yu JJ, Deng SL, Huang H et al (2023) Exploring the potential applications of the noninvasive reporter gene in plant genetic transformation. Forests 14(3):637. 10.3390/f14030637

[CR34] Zhang Y, Liang Z, Zong Y et al (2016) Efficient and transgene-free genome editing in wheat through transient expression of CRISPR/Cas9 DNA or RNA. Nat Commun 7:12617. 10.1038/ncomms12617 27558837 10.1038/ncomms12617 PMC5007326

[CR35] Zhang Y, Restall J, Crisp P et al (2021) Current status and prospects of plant genome editing in Australia. In Vitro Cell Dev Biol Plant 57:574–583. 10.1007/s11627-021-10188-y 34054265 10.1007/s11627-021-10188-y PMC8143062

