# Supplementary material for: An accelerated transgene-free genome editing system using microparticle bombardment of sorghum immature embryos
Source: aBIOTECH. 2025 Mar 4;6(2):202–14. doi: 10.1007/s42994-025-00204-9 (PMC12238444; doi:10.1007/s42994-025-00204-9)
Supplement: Supplementary file 1 — Supplementary file1 (DOCX 39 KB) [file 42994_2025_204_MOESM1_ESM.docx]

**Online Resource Supplementary materials**

Supplementary Table 1 Editing events of all achieved albino plants.

| Experiment and groups | No. | Sequencing results |
| --- | --- | --- |
|  |  |  |
| Experiment 1 | | |
| Selective  Group (SRM) | 1 | Large deletion |
|  | 2 | Large deletion |
|  | 3 | Complex rearrangement |
|  | 4 | Complex rearrangement |
|  | 5 | Large deletion |
|  | 6 | Large deletion |
|  | 7 | Complex rearrangement |
|  | 8 | Small indels: One nucleotide insertion and Cas9 insertion |
|  | 9 | Large deletion |
|  | 10 | Large deletion |
|  | 11 | Large deletion |
|  | 12 | Large deletion |
|  | 13 | Large deletion |
|  | 14 | Complex rearrangement |
|  | 15 | Large deletion |
|  | 16 | Large deletion |
| Selection-free Group | 1 | Complex rearrangement |
|  | 2 | Large deletion |
|  | 3 | Large deletion |
|  | 4 | Large deletion |
|  | 5 | Large deletion |
|  | 6 | Large deletion |
|  | 7 | Small indels: 2 nucleotides insertion |
|  | 8 | Complex rearrangement |
|  | 9 | No change compared to Tx430 |
|  | 10 | Large deletion |
|  | 11 | Complex rearrangement |
|  | 12 | Complex rearrangement |
|  | 13 | Large deletion |
|  | 14 | Large deletion |
|  | 15 | Complex rearrangement |
|  | 16 | Complex rearrangement |
|  | 17 | Large deletion |
|  | 18 | Complex rearrangement |
| Experiment 2 | | |
| Selective Group | 1 | Small indels: 1: G-A, 2: insertion |
|  | 2 | Complex rearrangement |
|  | 3 | Large deletion |
|  | 4 | Complex rearrangement & part of deletion |
|  | 5 | Small indels: 1: - 4nt, 2: + 1nt |
|  | 6 | Complex rearrangement |
|  | 7 | Small indels: 1: - 4nt, 2: +1nt |
|  | 8 | 1: Complex rearrangement, 2: + 2nt |
|  | 9 | Complex rearrangement |
|  | 10 | Small indels: 1: - 14nt, 2: - 15nt |
|  | 11 | Large deletion |
|  | 12 | Complex rearrangement |
|  | 13 | Complex rearrangement |
|  | 14 | No change compared to Tx430 |
|  | 15 | Small indels: 1: - 14nt, 2: +1nt |
|  | 16 | Small indels: 1: -14nt, 2: +1nt |
|  | 17 | Complex rearrangement |
|  | 18 | Complex rearrangement |
|  | 19 | Complex rearrangement |
|  | 20 | Complex rearrangement & small Intermittent insertion |
|  | 21 | Large deletion |
|  | 22 | Small indels: 1: +1nt, 3nt substitution |
|  | 23 | Large deletion+ complex rearrangement |
|  | 24 | Large deletion |
|  | 25 | Complex rearrangement |
|  | 26 | Complex rearrangement |
|  | 27 | Complex rearrangement |
|  | 28 | Complex rearrangement |
|  | 29 | Complex rearrangement |
|  | 30 | Complex rearrangement and unknown insertion (highly similar to some part of Cas9 plasmid) |
|  | 31 | Small indels: 1: -4nt, 2:+1nt |
|  | 32 | No change compared to Tx430 |
|  | 33 | Large deletion |
|  | 34 | 1: large deletion, 2: NPTII insertion |
| Selection-free  Group | 1 | Complex rearrangement |
|  | 2 | Complex rearrangement |
|  | 3 | N/A |
|  | 4 | Complex rearrangement |
|  | 5 | Small indels: 1: +1nt, 2: -1nt and 1nt change |
|  | 6 | Intermittent deletion |
|  | 7 | No change compared to Tx430 |
|  | 8 | No change compared to Tx430 |
|  | 9 | Intermittent deletion (BlpR & Cas9 from zCas9 plasmid) |
|  | 10 | Complex rearrangement |
|  | 11 | 1: Complex rearrangement, 2: ~200nt AmpR insertion form NPTII plasmid |
|  | 12 | Large deletion |
|  | 13 | No change compared to Tx430 |
|  | 14 | Complex rearrangement |
|  | 15 | Complex rearrangement |
|  | 16 | Large deletion |
|  | 17 | Complex rearrangement |
|  | 18 | Small indels: 1: no change, 2: +1nt |
|  | 19 | Complex rearrangement |
|  | 20 | Complex rearrangement |
|  | 21 | 1: Complex rearrangement 2: ~500bp Cas9 insertion from Cas9 plasmid |

Supplementary Table 2 Summary of PCR results for six primer pairs targeting zCas9 and *SbPDS_gRNA* plasmids in Experiment 1 and Experiment 2

| Experiment and groups | No. | zCas9 plasmid | | | SbPDS_gRNA plasmid | | | Transgene-free (yes/No) |
| --- | --- | --- | --- | --- | --- | --- | --- | --- |
|  |  | Cas 9 | Ubi | Ori | NPT2 | Amp | gRNA |  |
| Experiment 1 | | | | | | | | |
| Selective Group (SRM) | 1 | + | + | + | + | + | **-** | No |
|  | 2 | + | + | + | + | + | **-** | No |
|  | 3 | + | + | + | + | + | + | No |
|  | 4 | + | + | + | + | + | + | No |
|  | 5 | + | + | + | **-** | **-** | **-** | No |
|  | 6 | + | + | + | + | + | + | No |
|  | 7 | + | + | + | + | **-** | + | No |
|  | 8 | + | + | + | + | + | + | No |
|  | 9 | + | + | + | + | + | + | No |
|  | 10 | + | + | + | **-** | **-** | **-** | No |
|  | 11 | + | + | + | + | + | + | No |
|  | 12 | + | + | + | + | + | + | No |
|  | 13 | + | + | + | + | + | + | No |
|  | 14 | - | + | + | + | + | + | No |
|  | 15 | + | + | + | + | **-** | + | No |
|  | 16 | + | + | + | + | + | + | No |
| Selection-free Group (RM) | 1 | - | - | - | - | - | - | Yes |
|  | 2 | + | - | - | - | - | - | No |
|  | 3 | + | + | + | - | - | - | No |
|  | 4 | + | + | + | - | - | - | No |
|  | 5 | + | + | + | - | + | + | No |
|  | 6 | + | - | - | - | - | - | No |
|  | 7 | + | - | - | - | - | - | No |
|  | 8 | + | - | - | - | - | - | No |
|  | 9 | - | - | - | - | - | - | Yes |
|  | 10 | + | - | + | - | - | - | No |
|  | 11 | - | - | - | - | - | - | Yes |
|  | 12 | + | - | - | - | - | - | No |
|  | 13 | - | - | - | - | - | - | Yes |
|  | 14 | + | + | + | + | + | + | No |
|  | 15 | + | + | + | - | - | - | No |
|  | 16 | + | - | - | - | - | - | No |
|  | 17 | + | + | + | - | - | - | No |
|  | 18 | + | - | - | - | - | - | No |
| Experiment 2 | | | | | | | | |
| Selective Group (SRM) | 1 | + | + | + | - | + | + | No |
|  | 2 | + | + | + | - | + | + | No |
|  | 3 | + | + | + | + | + | + | No |
|  | 4 | + | + | - | - | - | - | No |
|  | 5 | + | + | - | - | - | - | No |
|  | 6 | + | + | - | - | - | - | No |
|  | 7 | + | + | - | - | - | - | No |
|  | 8 | - | - | - | - | - | - | Yes |
|  | 9 | + | + | + | + | + | + | No |
|  | 10 | + | + | + | + | + | + | No |
|  | 11 | + | + | - | + | + | - | No |
|  | 12 | + | + | + | + | + | - | No |
|  | 13 | + | + | - | - | - | - | No |
|  | 14 | + | + | - | - | - | - | No |
|  | 15 | + | + | + | - | + | + | No |
|  | 16 | + | + | - | - | - | - | No |
|  | 17 | + | + | - | - | - | - | No |
|  | 18 | + | + | + | + | + | + | No |
|  | 19 | + | + | - | - | - | - | No |
|  | 20 | + | + | + | - | + | + | No |
|  | 21 | + | + | + | - | + | + | No |
|  | 22 | + | - | - | - | - | - | No |
|  | 23 | + | + | + | + | + | + | No |
|  | 24 | + | + | + | + | + | + | No |
|  | 25 | + | - | - | + | + | - | No |
|  | 26 | - | - | - | - | - | - | Yes |
|  | 27 | + | + | + | - | - | + | No |
|  | 28 | + | + | + | - | + | + | No |
|  | 29 | + | + | + | - | - | + | No |
|  | 30 | + | + | - | - | + | + | No |
|  | 31 | + | - | - | - | - | - | No |
|  | 32 | + | + | - | - | - | - | No |
|  | 33 | + | + | - | - | + | - | No |
|  | 34 | + | + | - | - | + | + | No |
| Selection-free Group (RM) | 1 | + | + | - | - | - | - | No |
|  | 2 | + | + | + | + | + | + | No |
|  | 3 | + | + | + | + | + | + | No |
|  | 4 | - | - | - | - | - | - | Yes |
|  | 5 | - | - | - | - | - | - | Yes |
|  | 6 | - | - | - | - | - | - | Yes |
|  | 7 | + | + | - | - | - | - | No |
|  | 8 | - | - | - | - | - | - | No |
|  | 9 | + | + | + | + | + | + | No |
|  | 10 | - | - | - | - | - | - | Yes |
|  | 11 | + | + | + | - | - | - | No |
|  | 12 | + | - | - | - | - | - | No |
|  | 13 | + | + | - | - | - | - | No |
|  | 14 | - | - | - | - | - | - | Yes |
|  | 15 | + | - | - | - | - | - | No |
|  | 16 | + | + | + | + | + | + | No |
|  | 17 | - | - | - | - | - | - | Yes |
|  | 18 | + | + | - | - | - | - | No |
|  | 19 | + | - | - | - | - | - | No |
|  | 20 | - | - | - | - | - | - | Yes |
|  | 21 | - | - | - | - | - | - | Yes |
